# Antiadherent and antibacterial properties of stainless steel and NiTi orthodontic wires coated with silver against *Lactobacillus acidophilus*—an in vitro study

**DOI:** 10.1186/s40510-015-0110-0

**Published:** 2015-11-17

**Authors:** Arun Rameshwar Mhaske, Pradeep Chandra Shetty, N. Sham Bhat, C. S. Ramachandra, S. M. Laxmikanth, Kiran Nagarahalli, Pawankumar Dnyandeo Tekale

**Affiliations:** Department of Orthodontics and Dentofacial Orthopedics, DY Patil Dental School, Lohegaon, Pune, Maharashtra India; Department of Orthodontics and Dentofacial Orthopedics, AECS Maaruti College of Dental Sciences and Research Centre, Bangalore, India; Department of Orthodontics, Dr Rajesh Ramdasji Kambe Dental College and Hospital, Akola, Maharashtra India

**Keywords:** Orthodontic wires, Antiadherent properties, Antibacterial properties, Silver coating, *Lactobacillus acidophilus*

## Abstract

**Background:**

The purpose of the study is to assess the antiadherent and antibacterial properties of surface-modified stainless steel and NiTi orthodontic wires with silver against *Lactobacillus acidophilus*.

**Methods:**

This study was done on 80 specimens of stainless steel and NiTi orthodontic wires. The specimens were divided into eight test groups. Each group consisted of 10 specimens. Groups containing uncoated wires acted as a control group for their respective experimental group containing coated wires. Surface modification of wires was carried out by the thermal vacuum evaporation method with silver. Wires were then subjected to microbiological tests for assessment of the antiadherent and antibacterial properties of silver coating against *L. acidophilus*. Mann–Whitney *U* test was used to analyze the colony-forming units (CFUs) in control and test groups; and Student’s *t* test (two-tailed, dependent) was used to find the significance of study parameters on a continuous scale within each group.

**Results:**

Orthodontic wires coated with silver showed an antiadherent effect against *L. acidophilus* compared with uncoated wires. Uncoated stainless steel and NiTi wires respectively showed 35.4 and 20.5 % increase in weight which was statistically significant (*P* < 0.001), whereas surface-modified wires showed only 4.08 and 4.4 % increase in weight (statistically insignificant *P* > 0.001). The groups containing surface-modified wires showed statistically significant decrease in the survival rate of *L. acidophilus* expressed as CFU and as log of colony count when compared to groups containing uncoated wires. It was 836.60 ± 48.97 CFU in the case of uncoated stainless steel whereas it was 220.90 ± 30.73 CFU for silver-modified stainless steel, 748.90 ± 35.64 CFU for uncoated NiTi, and 203.20 ± 41.94 CFU for surface-modified NiTi.

**Conclusions:**

Surface modification of orthodontic wires with silver can be used to prevent the accumulation of dental plaque and the development of dental caries during orthodontic treatment.

## Background

Enamel demineralization, or white spot lesions around orthodontic appliances, is a common side effect of orthodontic treatment. The oral environment with fixed appliance provides ideal conditions for colonization of microorganisms as a result of their inherent morphologic irregularities [1]. The resultant increase in plaque accumulation and retention areas places the patient at higher risk for enamel demineralization adjacent to the appliance and exacerbates the effects of preexisting incipient carious lesions. The incidence of enamel demineralization and periodontal disease after fixed orthodontic treatment can involve up to 50 % of patients. The incidence of such white spot lesions around orthodontic brackets has been demonstrated within 1 month of treatment [2, 3].

Among different fixed orthodontic appliances, wires could play a significant role in enamel demineralization because they are present throughout the period of orthodontic treatment. Areas of contact between the wire and brackets provide a unique environment that impedes proper access to tooth surfaces for cleaning. In a study done by Eliades et al. [4], it was seen that stainless steel represented the highest critical surface tension and energy and can be expected to have higher plaque retaining capacity.

In addition, stainless steel was found to induce specific changes in the oral environment such as decreased pH, increased plaque accumulation [1, 5, 6], and elevated *Streptococcus mutans* and *Lactobacillus acidophilus* colonization [7–10]. Among several pathogenic organisms that accumulate and colonize in the form of plaque, lactobacilli do not play a major part in initiation but are important in progression of the lesion. With established low pH, the number of lactobacilli increases and the number of *S. mutans* decreases; this contributes to demineralization of the teeth once lesions are established. Preventing these lesions is an important concern for the orthodontist, because the lesions are unaesthetic, unhealthy, and potentially irreversible.

With the emergence of antibiotic-resistant strain of bacteria, certain metals particularly in nanoparticle form have attracted attention. Nanoparticles are insoluble particles having size smaller than 100 nm. Bacteria are less likely to develop resistance to metal nanoparticles as compared to conventional antibiotics. Nanoparticles can be used either combining with dental materials or by coating the surface which aims to reduce the microbial adhesion and prevent caries [11].

Among the various metals, silver since ages is known for its antimicrobial activity against Gram-positive and -negative bacteria, fungi, protozoa, and certain viruses, including antibiotic-resistant strains. Because of these properties, silver is widely used in burned areas, medical devices, textile fabric, and as a water purifier [12]. Surface coating of silver can be obtained by different methods, e.g., physical vapor deposition, electrodeposition, electroless, and metallurgical [13]. Certain silver thin films prepared by physical vapor deposition exhibit a strong antimicrobial effect as compare to others [14].

Various studies have demonstrated the effect of silver nanoparticles on multiple organisms [11, 14–16]. But there are few studies where the orthodontic wires are modified with silver and tested for its antimicrobial properties. Thus, our study was designed to evaluate the antiadherent and antibacterial properties of stainless steel and NiTi orthodontic wires modified with silver coating against *L. acidophilus*.

## Methods

This study was done on 80 specimens of orthodontic wires each of 5 cm in length. The specimens were divided into eight test groups. Each group consisted of 10 specimens. The groups containing uncoated stainless steel and uncoated nickel titanium wires acted as control group for their respective experimental group containing coated stainless steel and nickel titanium wires (Tables 1 and 2).

**Table 1:** Groups of wires used for antiadherent property of surface-modified orthodontic wires

Group 1	Control group—it consisted of 10 uncoated stainless steel orthodontic wires
Group 2	Experimental group—it consisted of 10 surface-modified stainless steel orthodontic wires coated with silver
Group 5	Control group—it consisted of 10 uncoated nickel titanium wires
Group 6	Experimental group—it consisted of 10 surface-modified nickel titanium orthodontic wires coated with silver

**Table 2:** Groups of wires used for antibacterial property of surface-modified orthodontic wires

Group 3	Control group—it consisted of 10 uncoated stainless steel orthodontic wires
Group 4	Experimental group—it consisted of 10 surface-modified stainless steel orthodontic wires coated with silver
Group 7	Control group—it consisted of 10 uncoated nickel titanium wires
Group 8	Experimental group—it consisted of 10 surface-modified nickel titanium orthodontic wires coated with silver

### Bacterial strains

*L. acidophilus* (NCL, Pune) strains were used for adhesion and viability tests. Lactobacilli were inoculated into 5 mL of MRS broth and were incubated for 24 h at 37 °C. For the adhesion test, 10 % of an overnight-cultured broth was transferred to 10 mL of the MRS broth containing 10 % sucrose and was incubated for 24 h.

### Preparation of silver-coated orthodontic wire

**Fig. 1:**
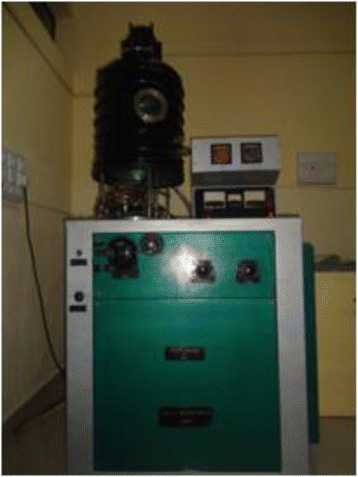
Vacuum coating equipment

**Fig. 2:**
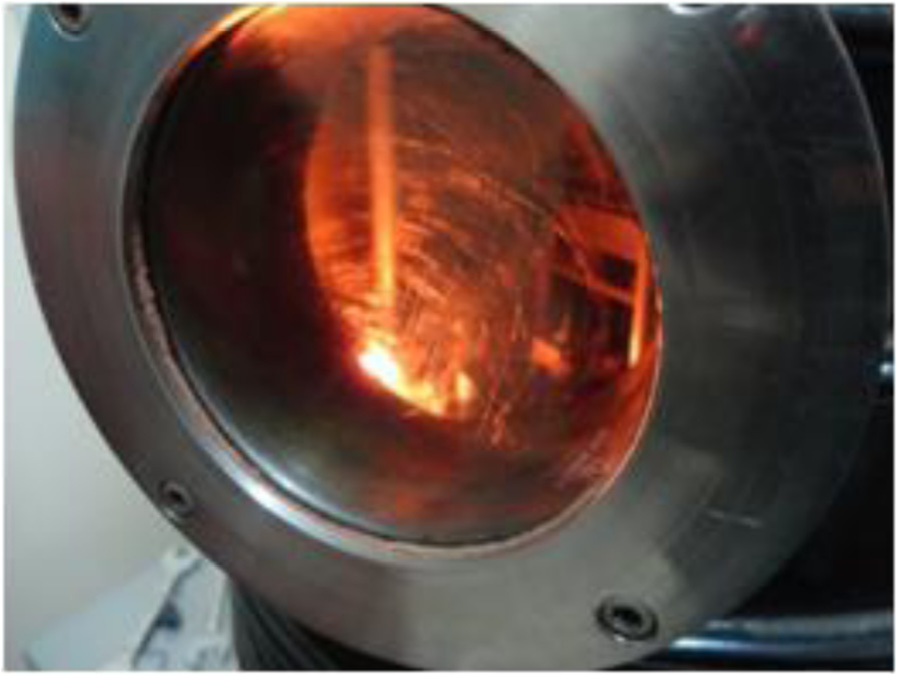
Coating process

Surface modification of both stainless steel and nickel titanium orthodontic wires with silver was carried out by thermal evaporation method. The instrument used for coating is HINDHIVAC vacuum coating unit model no-15 F6 (Hind High Vacuum Co. Bangalore) (Fig. 1) at IISC, Bangalore. It produces thin homogenous, uniform pure film coating of various metals to achieve controlled effects in various applications (Fig. 2). Pure silver (99.9 %) was used to obtain thin coating on orthodontic wire. Silver was heated through its vaporization temperature in a closed chamber and vapors were allowed to pass through a valve which could be controlled according to the desired thickness. In this study, 10-nm-thick silver film was coated on orthodontic wires to avoid any significant change in its dimension.

### Evaluation of bacterial adhesion to orthodontic wires

Before the adhesion test, ultrasonication of the wires were done for 5 min in 2-propanol to remove adventitious macroscopic contaminations and were dried in a desiccator. After cleaning and sterilizing in an autoclave, the wires were preweighted using an analytical balance (Xitij Instrument, Pune) and were stored in an airtight container.

**Fig. 3:**
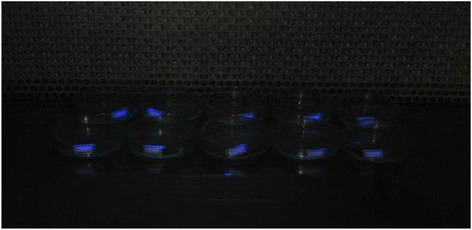
Incubator

In a sterile beaker containing 10 mL of an MRS broth, an overnight-cultured lactobacillus culture broth was inoculated to a final concentration of 10 %. After this, 1 mL of this suspension was pipetted into each of the tubes, and the wires were immersed in it and were incubated for 24 h at 37 °C inside the laminar air flow chamber or inoculators (Fig. 3). Wires to which bacteria adhered were carefully removed and immersed in a 10 % formaldehyde solution for 30 min to immobilize the cells. After a careful rinse with distilled water, the wires were dried in a desiccator for 24 h. The weight change of the brackets during the bacterial adhesion test was recorded with an analytical balance.

### Antibacterial activity assay of orthodontic wires

**Fig. 4:**
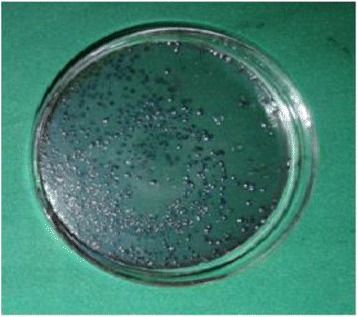
Colonies of *L. acidophilus*

Antibacterial activities of the surface-modified orthodontic wires were demonstrated against lactobacilli. First, the lactobacillus culture broth was diluted with MRS broth to achieve an optical density of 1.0 at 660 nm. Ten milliliters of the diluted bacterial suspension was transferred onto petri dishes containing uncoated and silver-coated wires. These dishes were incubated inside the laminar air flow chamber. After incubation, 100 mL of the bacterial suspension was serially diluted and plated onto MRS agar plates. Antibacterial activity was described as the survival rate by colony-forming units (CFUs) for lactobacilli (Fig. 4).

## Statistical software

Statistical software, namely, SAS 9.0 (SAS Institute Inc, Cary, NC), the Statistical Package for the Social Sciences (SPSS), version 15.0 (SPSS Inc, Chicago, IL), Stata 8.0 (Stata Corp, College Station, TX), MedCalc 9.0.1 (MedCalc Software bvba, Mariakerke, Belgium), and Systat 11.0 (Systat Software Inc, Chicago, IL), was used for analysis of data; Microsoft Word and Excel were used to generate graphs and tables; Mann–Whitney *U* test was used to analyze the CFUs in control and test groups; and Student’s *t* test (two-tailed, dependent) was used to find the significance of study parameters on a continuous scale within each group.

## Results

### Adhesion of *L. acidophilus* to the surface of orthodontic wires

From the data shown in Table 3, it is seen that Group 1 had initial average weight of 0.24 ± 0.021 and final average weight of 0.325 ± 0.035 with the increase in weight being in range of 0.085 ± 0.024, which was statistically significant (*P* < 0.001).

**Table 3:** Comparison of weight: initial, final, and change in weight of wires

Group	Weight			Significance
	Initial	Final	Change	
Group 1	0.240 ± 0.021	0.325 ± 0.035	0.085 ± 0.024	*P* < 0.001**
	(0.200–0.250)	(0.250-0.350)	(35.4 %)	
Group 2	0.245 ± 0.028	0.255 ± 0.037	0.010 ± 0.020	*P* = 0.168
	(0.200–0.300)	(0.200–0.300)	(4.08 %)	
Group 5	0.220 ± 0.026	0.265 ± 0.033	0.045 ± 0.028	*P* < 0.001**
	(0.200–0.250)	(0.250–0.350)	(20.5 %)	
Group 6	0.225 ± 0.042	0.235 ± 0.047	0.010 ± 0.021	*P* = 0.169
	(0.150–0.300)	(0.150–0.300)	(4.4 %)	

**Statistically significant

Group 2 had an initial average weight of 0.245 ± 0.028 and a final average weight of 0.255 ± 0.037 with the increase in weight being in range of 0.010 ± 0.020, which was statistically insignificant (*P* > 0.001).

It can be seen that uncoated stainless steel wires showed 35.4 % increase in weight whereas surface-modified wires showed only 4.08 % increase in weight.

Group 5 had an initial average weight of 0.220 ± 0.026 and a final average weight of 0.265 ± 0.033 with the increase in weight being in range of 0.045 ± 0.028, which was statistically significant (*P* < 0.001).

Group 6 had an initial average weight of 0.225 ± 0.042 and a final average weight of 0.235 ± 0.047 with the increase in weight being in range of 0.010 ± 0.021, which was statistically insignificant (*P* > 0.001).

It can be seen that uncoated nickel titanium wires showed 20.5 % increase in weight whereas surface-modified wires showed only 4.4 % increase in weight.

Thus, uncoated orthodontic wires showed statistically significant increase in the weight when compared to the surface-modified orthodontic wires (Table 3).

### Antibacterial activity of surface-modified orthodontic wires on *L. acidophilus*

The groups that were used to assess the adhesion of *L. acidophilus* to the surface of orthodontic wires are as shown in Table 2. Survival rate of the bacterial cells is calculated in terms of CFUs.

In Table 4, it is seen that in the dilution agar plate method, the survival rate of *L. acidophilus* was 836.60 ± 48.97 CFU in the case of Group 3 whereas it was 220.90 ± 30.73 CFU in Group 4.

**Table 4:** Comparison of colony count

Group	Colony count		*P* value
	Range	Mean ± SD	
Group 3	776–934	836.60 ± 48.97	*P* < 0.001**
Group 4	176–262	220.90 ± 30.73	
Group 7	710–810	748.90 ± 35.64	*P* < 0.001**
Group 8	117–264	203.20 ± 41.94	

**Statistically significant

Similarly, the survival rate of *L. acidophilus* was 748.90 ± 35.64 CFU in the case of Group 7 whereas it was 203.20 ± 41.94 CFU in case of Group 8.

Thus, the groups containing surface-modified wires showed statistically significant decrease in the survival rate of *L. acidophilus* expressed as CFU and as log of colony count when compared to groups containing uncoated wires (Table 4).

## Discussion

From the time when the symbiotic association between *S. mutans* and *L. acidophilus* was first established as causative factor for caries, many methods attempted to prevent colonization of these microorganisms. Now, it is well established that *S. mutans* is responsible for caries initiation whereas *L. acidophilus* is responsible for the progression of caries [10, 11]. With the progression of the caries, the number of streptococcus decreases and that of lactobacillus increases. Most of the studies in the literature using silver as an antimicrobial agent concentrate on the streptococcus (aerobic bacteria) [10, 11, 14, 15]; this study proves that silver is also effective against anaerobic bacteria, i.e., *Lactobacillus*.

Silver has an important microbial effect. The interaction of silver with thiol groups in enzymes and proteins plays an essential role in its antimicrobial action, although other cellular components, like hydrogen bonding, may also be involved [17]. Silver has been proposed to act by binding to key functional groups of enzymes. It also causes the release of K^+^ ions from bacterial plasma or cytoplasmic membrane, which is a site associated with many important bacterial enzymes, thus making it an efficient target site for silver action [17].

In recent studies, surface modification of stainless steel orthodontic wires and brackets with photocatalytic TiO_2_ and TiAg (titanium silver) has led to positive results [18–20]. But it has some disadvantages which includes change in color of wires and brackets after TiO_2_ coating and also if coating is done on NiTi wires, it is not advisable to heat them at 500 °C for 5 h, which may lead to loss of its properties.

In the present study, the surfaces of orthodontic wires were coated with silver without heating and minimal changes in its color were observed. Our findings showed that silver-coated wires have effective antiadherent properties against *L. acidophilus*. Also, the number of bacteria determined by conventional plate counting, which counts only culturable colonies in media, was significantly lower than the control. These results concluded that silver nanoparticles may be one of the most effective for controlling *L. acidophilus* reducing the progression of caries. Size reduction of silver in nanoparticle form is an important condition for the effect of silver. Smaller size provides greater surface-to-volume ratio, leading to more close interaction with microbial membrane and larger surface area for antimicrobial activity [11].

However, the use of silver must be undertaken with caution, since the concentration-dependent toxicity has been demonstrated. Silver has not been mentioned in the list of the hazardous heavy metals to public health but still accumulation in the environment should be considered [12]. The amount silver essential to successfully carry out antiadherent, antibacterial properties and the maximum lethal dose should be determined if its toxicity is to be avoided before it can be applied to orthodontic materials. The silver coating on wires is purely surface based and may be prone to wear during archwire sliding. Thus, it is critical to assess the durability and sustainability of silver coatings under clinical situations in the oral environment. Also, it would be prudent to determine whether the thin coating of silver would alter its mechanical properties.

## Conclusions

- The silver coating prevented the adhesion of *L. acidophilus* to the orthodontic wires, hence demonstrating its antiadherent properties.
- The silver coating also demonstrated antibacterial effects against *L. acidophilus*.
- Surface modification of orthodontic wires with silver can be used to prevent the development of dental plaque and dental caries during orthodontic treatment.

## References

[1] Balenseifen JW, Madonia JV (1970). Study of dental plaque in orthodontic patients. J Dent Res.

[2] Gorelick L, Geiger AM, Gwinnett AJ (1982). Incidence of white spot formation after bonding and banding. Am J Orthod.

[3] Artun J, Brobakken B (1986). Prevalence of caries and white spot lesions after orthodontic treatment with multibanded appliance. Eur J Orthod.

[4] Eliades T, Eliades G, Brantley WA (1995). Microbial attachment on orthodontic appliances. I. Wettability and early pellicle formation on bracket materials. Am J Orthod Dentofacial Orthop.

[5] Menzaghi N, Saletta M, Garattini G, Brambilla E, Strohmenger L (1991). Changes in the yeast oral flora in patients in orthodontic treatment. Prev Assist Dent.

[6] Chatterjee R, Kleinberg I (1979). Effect of orthodontic band placement on the chemical composition of human incisor tooth plaque. Arch Oral Biol.

[7] Rosenbloom RG, Tinanoff N (1991). Salivary S. mutans levels in patients before, during, and after orthodontic treatment. Am J Orthod Dentofacial Orthop.

[8] Scheie AA, Arnesberg P, Krogstad O (1984). Effects of orthodontic treatment on prevalence of Streptococcus mutans in plaque and saliva. Scand J Dent Res.

[9] Mattingly JA, Sauer GJ, Yancey JM, Arnold RR (1983). Enhancement of S. mutans colonization by direct bonded orthodontic appliances. J Dent Res.

[10] Sug-Joon A, Bum-Soon L, Shin-Jae L (2007). Prevalence of cariogenic streptococci on incisor brackets detected by polymerase chain reaction. Am J Orthod Dentofacial Orthop.

[11] Borzabadi-Farahani A, Borzabadi E, Lynch E (2014). Nanoparticles in orthodontics, a review of antimicrobial and anti-caries application. Acta Odontol Scand.

[12] Douglas RM, Luiz FG, Aline ST, Adhemar CR, Emerson RC, Debora BB (2009). The growing importance of material that prevent microbial adhesion: antimicrobial effect of medical devices containing silver. Int J Antimicrob Agents.

[13] Djokic SS, Burrell RE (1998). Behavior of silver in physiologic solutions. J Electrochem Soc.

[14] Yamamoto K, Ohashi S, Aono M, Kokubo T, Yamada I, Yamauchi J (1996). Antibacterial activity of silver ions implanted in SiO_2_ filler on oral streptococci. Dent Mater.

[15] Hernandez-Sierra JF, Ruiz F, Pena DC, Martinez-Gutierrez F, Martinez AE, Guillen Ade J (2008). The antimicrobial sensitivity of Streptococcus mutans to nanoparticles of silver, zinc oxide and gold. Nanomedicine.

[16] Balazs DJ, Triandafillu K, Wood P, Chevolot Y, Van Delben C, Harms H (2004). Inhibition of bacterial adhesionon PVC endotracheal tube by RF oxygen glow discharge, sodium hydroxide and silver nitrate treatments. Biomaterials.

[17] Woo KJ, Hye CK, Ki WK, Shin S, Kim SH, Yong HP (2008). Antibacterial activity and mechanism of action of the silver ion in *Staphylococcus aureus* and *Escherichia coli*. Appl Environ Microbiol.

[18] Choi JY, Chung CJ, Oh KT, Choi YJ, Kim KH (2009). Photocatalytic antibacterial effect of TiO2 film of TiAg on Streptococcus mutans. Angle Orthod.

[19] Chun MJ, Shim E, Kho EH, Park KJ, Jung J, Kim JM (2007). Surface modification of orthodontic wires with photocatalytic titanium oxide for its antiadherent and antibacterial properties. Angle Orthod.

[20] Shah AG, Shetty PC, Ramachandra CS, Bhat NS, Laxmikanth SM (2011). In vitro assessment of photocatalytic titanium oxide surface modified stainless steel orthodontic brackets for antiadherent and antibacterial properties against lactobacillus acidophilus. Angle Orthodontist.

